# Focus on: The Burden of Alcohol Use—Trauma and Emergency Outcomes

**DOI:** 10.35946/arcr.v35.2.05

**Published:** 2014

**Authors:** Cheryl J. Cherpitel

**Affiliations:** **Cheryl J. Cherpitel, Dr.P.H.***is a senior scientist in the Alcohol Research Group, Emeryville, California.*

**Keywords:** Alcohol consumption, alcohol-related injury, alcohol and drug related–injury, alcohol-attributable fractions, risk factors, alcohol and other drug–induced risk, hospital, emergency department, emergency room, emergency care, trauma, injury, intentional injury, unintentional injury, patients, case–control studies, case–crossover studies

## Abstract

Hospital emergency departments (EDs) see many patients with alcohol-related injuries and therefore frequently are used to assess the relationship between alcohol consumption and injury risk. These studies typically use either case–control or case–crossover designs. Case–control studies, which compare injured ED patients with either medical ED patients or the general population, found an increased risk of injury after alcohol consumption, but differences between the case and control subjects partly may account for this effect. Case–crossover designs, which avoid this potential confounding factor by using the injured patients as their own control subjects, also found elevated rates of injury risk after alcohol consumption. However, the degree to which risk is increased can vary depending on the study design used. Other factors influencing injury risk include concurrent use of other drugs and drinking patterns. Additional studies have evaluated cross-country variation in injury risk as well as the risk by type (i.e., intentional vs. unintentional) and cause of the injury. Finally, ED studies have helped determine the alcohol-attributable fraction of injuries, the causal attribution of injuries to drinking, and the impact of others’ drinking. Although these studies have some limitations, they have provided valuable insight into the association between drinking and injury risk.

Alcohol consumption is a leading risk factor for morbidity and mortality related to both intentional (i.e., violence-related) and unintentional injury. In 2000, 16.2 percent of deaths and 13.2 percent of disability-adjusted life years (DALYs) from injuries, worldwide, were estimated to be attributable to alcohol (Rehm et al. 2009). Alcohol affects psychomotor skills, including reaction time, as well as cognitive skills, such as judgment; as a result, people drinking alcohol often place themselves in high-risk situations for injury.

Much of the data linking alcohol with nonfatal injuries have come from studies conducted in hospital emergency departments (EDs). As described in this article, in these settings the prevalence of alcohol involvement in the patients’ injuries, as measured by a positive blood alcohol concentration (BAC) at the time of arrival in the ED or self-reported drinking prior to the injury event, is substantial. To accurately assess the relationship between alcohol use and injury risk, ED studies generally have used probability sampling designs, in which all times of day and days of the week are represented equally. This approach circumvents biases associated with sampling that might occur, for example, if samples were identified only on weekend evenings, when a higher prevalence of drinking and, possibly, of injury might be expected. Although the high prevalence rates mentioned above suggest that alcohol is an important risk factor for injury, they do not provide the information necessary to evaluate the actual level of risk for injury at which drinking places the individual.

Data to establish drinking-related risk of both intentional and unintentional injury in ED samples generally have come from two types of study design: case–control studies and case–crossover studies. This article summarizes the findings of these studies and explores specific aspects of the relationship between alcohol use and injury risk.

## Risk of Injury in ED Studies

### Case–Control Studies

Two types of case–control studies have been used to estimate the risk of injury from drinking for patients treated in the ED. The most commonly used type of case–control study uses noninjured (i.e., medical) patients attending the same ED during the same period of time as quasi-control subjects. These patients presumably come from the same geographic area as the injured patients and likely share other characteristics (e.g., socioeconomic status). Researchers conducted a meta-analysis of 15 ED studies conducted in 7 countries that participated in the Emergency Room Collaborative Alcohol Analysis Project (ERCAAP) (Cherpitel et al. 2003*a*) and which all used the same methodology and instrumentation. The studies only included those patients who arrived at the ED within 6 hours of the injury event and excluded those medical patients who primarily were admitted to the ED for alcohol intoxication or withdrawal symptoms. The meta-analysis found a pooled odds ratio (OR) of injury associated with a positive BAC (≥0.01 percent) of 2.4 (95% CI = 1.9–3.0);^1^ moreover, the OR was higher (OR = 2.9) for patients with higher BAC levels (≥0.10 percent) (Ye and Cherpitel 2009). A similar likelihood of injury (OR = 2.1) was found for patients who reported drinking within 6 hours prior to the injury event, regardless of time of arrival in the ED.

One concern with this approach of using medical patients as control subjects for injured patients is the possibility of underestimating the true risk of drinking associated with injury. Noninjured patients have been found to be heavier drinkers than people in the general population from which they come who do not seek emergency care (Cherpitel 1993). Thus, these patients may be attending the ED for conditions related to their drinking (in addition to those associated with alcohol intoxication or withdrawal).

In the second type of case–control study used to estimate risk of injury from drinking in ED patient samples, people in the general population of the community from which the ED patients come are used as control subjects. These individuals presumably are free of conditions that may be related to their drinking. Only four such studies have been reported to date, including two from Australia (Mcleod et al. 1999; Watt et al. 2004) and one each from the United States (Vinson et al. 2003) and Mexico (Borges et al. 1998). In these studies, the ORs ranged from 6.7 in the Mexican study to 3.1 in the U.S. study and around 2.0 in the Australian studies. Moreover, both the U.S. and the Australian studies demonstrated a dose-response relationship.

### Case–Crossover Studies

The second study design used to estimate the risk of injury from alcohol consumption is the case–crossover study (Maclure 1991). This approach is thought to circumvent at least some of the problems raised with the case–control design, such as demographic and others differences between case and control subjects that may be related to both alcohol consumption and likelihood of injury. There are two approaches to the case–crossover design, both of which use injured patients as their own control subjects, thereby theoretically reducing confounding of the alcohol–injury relationship from stable risk factors, such as age and gender.

- *The matched-interval approach.* Studies using the matched-interval approach compare drinking within 6 hours prior to the injury event with drinking during a predetermined control period, such as the same 6-hour period during the previous day or previous week. Such studies have reported ORs ranging from 3.2 (based on any drinking at the same time the previous day) (Vinson et al. 2003) to 5.7 in a 10-country study (based on any drinking at the same time the previous week) (Borges et al. 2006*b*). Both studies demonstrated a dose-response relationship. Thus, the analysis of Vinson and colleagues (2003) determined ORs ranging from 1.8 with consumption of 1 to 2 drinks prior to injury to 17 with consumption of 7 or more drinks. Likewise, Borges and colleagues (2006*b*) found ORs ranging from 3.3 with consumption of one to two drinks to 10.1 with consumption of six or more drinks prior to injury.
- *The usual-frequency approach.* This approach compares the patients’ drinking in the 6 hours preceding the injury to their expected drinking during that time, based on their usual frequency of drinking. In a study using this approach that included 28 EDs across 16 countries, the estimated ORs ranged from 1.05 (Canada) to 35.0 (South Africa), with a pooled estimate of 5.69 (95% CI = 4.04–8.00) (Borges et al. 2006*a*).

### Comparison of Methods to Estimate Risk

The results described above indicate that the estimates of risk of injury in samples from the same country can vary depending on the method used. For example, in analyses across eight countries participating in ERCAAP, analyses using the case–control method found that the pooled OR of injury for self-reported drinking prior to the event was 2.1, compared with an OR of 5.2 when the usual-frequency method of case–crossover analysis was used (Ye and Cherpitel 2009). Furthermore, the World Health Organization (WHO) Collaborative Study on Alcohol and Injury, which used the case–crossover method across 12 countries, found a pooled OR of injury of 6.8 using the usual-frequency approach, compared with 5.7 using the matched-interval approach (Borges et al. 2006*b*). Case–control designs may underestimate the risk of injury if noninjured control subjects are presenting to the ED with other conditions related to their drinking, whereas both the matched-interval and usual-frequency approaches to the case–crossover design are subject to recall bias of drinking in the past.

## Effects of Other Factors on Risk of Injury

### Effects of Other Drug Use

None of these estimates of risk of injury related to drinking have taken into consideration other drug use at the time of injury, although multiple substances commonly are used together in ED populations (Buchfuhrer and Radecki 1996). Other drug use might be expected to elevate the risk of injury, either alone or in combination with alcohol; however, this may not be the case. One study found an OR of 3.3 for drinking within 6 hours prior to injury and an OR of 3.0 for drinking in combination with other drug use during the same time; in contrast, drug use alone had no significant effect on risk (Cherpitel et al. 2012*b*). It is important to consider that in this study the majority of drug users reported using marijuana. However, given their different pharmacological properties, all drugs would not be expected to act in a similar manner, either alone or in combination with alcohol. Consequently, in other populations with different drug use patterns the findings might be different.

### Effects of Usual Drinking Patterns

The risk of injury from drinking prior to the event (i.e., acute consumption) also is influenced by the drinker’s usual drinking patterns (i.e., chronic consumption). Cherpitel and colleagues (2004) found that the risk of injury from drinking prior to the event was lower among frequent heavy drinkers than among infrequent heavy drinkers, suggesting that heavier drinkers may have developed tolerance against some adverse effects of alcohol that lead to injury. Likewise, in an analysis by Gmel and colleagues (2006), the risk of injury was greater among usual light drinkers who occasionally drink heavily (i.e., report episodic heavy drinking) than among people who usually drink heavily but report no episodic heavy drinking or among people who usually drink heavily as well as report episodic heavy drinking.

## Risk of Alcohol-Related Injury

Although acute alcohol consumption, modified by drinking pattern, has been found to be associated with risk of injury, drinking pattern also has been found to be associated with risk of an alcohol-related injury^2^ (defined as drinking within 6 hours prior to injury), with frequency of drinking among non–heavy drinkers (Cherpitel et al. 2003*b*) and both episodic and frequent heavy drinking predictive of alcohol-related injury (Cherpitel et al. 2012*c*). An analysis of combined data from ERCAAP and from the WHO Collaborative Study on Alcohol and Injury across 16 countries found the pooled risk of alcohol-related injury was increased with heavy episodic drinking (OR = 2.7) as well as with chronic high-volume drinking (OR = 3.5); moreover, the risk was highest for people reporting both patterns of drinking (OR = 6.1) (Ye and Cherpitel 2009).

## Cross-country Variation in Risk of Injury

A great deal of variation has been found across countries in risk of injury and risk of alcohol-related injury, and this heterogeneity seems to be associated with a country’s level of detrimental drinking pattern (DDP). The DDP score, which is based on aggregate survey data and key informant surveys, is a measure developed for comparative risk assessment in the WHO’s Global Burden of Disease study (Rehm et al. 2004). It includes such indicators of drinking patterns as heavy drinking occasions, drinking with meals, and drinking in public places. The DDP has been assessed in a large number of countries around the world as a measure of the “detrimental impact” on health, and other drinking-related harms, at a given level of alcohol consumption (Rehm et al. 2001, 2003). Countries with a higher level of DDP have been found to have a higher risk of injury related to alcohol than those with lower DDP scores (Cherpitel et al. 2005*b*).

## Risk by Type and Cause of Injury

Risk of injury from alcohol also varies by type (i.e., intentional vs. unintentional) and cause of injury. For example, Macdonald and colleagues (2006) found that the risk was highest for violence-related (i.e., intentional) injuries. A case–crossover analysis using the usual-frequency approach that included data from 15 countries in the ERCAAP and WHO projects found that greater variations across countries existed in risk of an intentional injury than in risk of unintentional injury; this difference was at least in part explained by the level of DDP in a country (Cherpitel and Ye 2010). Overall, the pooled OR for intentional injury related to drinking in these countries was 21.5, compared with 3.37 for unintentional injury (Borges et al. 2009). Furthermore, the risk of intentional injury showed a greater dose–response association than the risk of unintentional injury (Borges et al. 2009). Thus, the ORs for intentional injuries ranged from 11.14 for one to two drinks prior to injury to 35.57 for five or more drinks during this time, whereas the ORs for unintentional injuries ranged from 3.86 to 6.4, respectively. Among the unintentional injuries, the risk also varied depending on the cause of the injury. For example, the OR was 5.24 for traffic-related injuries, compared with 3.39 for injuries related to falls.

## Alcohol-Attributable Fraction

Another variable that has been studied in the context of assessing the risk of injuries after drinking is the alcohol-attributable fraction (AAF). This variable represents the proportional reduction in injury that would be expected if the risk factor (i.e., drinking prior to injury) was absent; it reflects the burden of injury in a given society that results from alcohol use. The AAF also varies across countries in ED studies, because it is related to both the risk of injury and the prevalence of alcohol-related injury. In a case–control study of 14 EDs from six countries in ERCAAP, the AAF based on self-reported drinking within 6 hours prior to the injury event varied from 0.5 percent to 18.5 percent for all types of injury, and from 19.1 percent to 83.3 percent for intentional injury (Cherpitel et al. 2005*a*). The pooled estimate from all EDs for the AAF was 5.8 percent for all types of injury and 42.5 percent for intentional injury. In other words, more than 40 percent of all intentional injuries would not have occurred if the people involved had not been drinking. Moreover, the investigators determined higher AAF estimates for male than female subjects for both unintentional injuries (5.5 percent vs. 1.7 percent) and intentional injuries (50.0 percent vs. 7.7 percent).

## Causal Attribution

The ED studies in the ERCAAP and WHO projects also assessed the patients’ causal attribution of their injuries to their drinking—that is, patients were asked whether they believed the injury would have occurred if they had not been drinking. In an evaluation that included 15 countries, one-half of the patients who reported drinking prior to injury also reported a causal attribution (Cherpitel et al. 2006). This information was used to establish a subjective AAF—an AAF derived from the patient’s own causal attribution of their injury to drinking. This subjective AAF then was compared to the AAF obtained using the standard formula based on the relative risk of injury from alcohol and prevalence of drinking in the 6-hour period (i.e., the objective AAF) from the six ERCAAP countries, as described above. This comparison found that for unintentional injuries, the subjective AAF generally was somewhat higher than the objective AAF. For intentional injuries, however, the subjective AAF was substantially lower (i.e., 5.9 percent to 46.7 percent) than the objective AAF (i.e., 24.9 percent to 83.3 percent) (Bond and Macdonald 2009).

## Others’ Drinking

Researchers also increasingly are interested in studying the harm, including injury, resulting from other people’s drinking. Evaluating these so-called externalities is important for a fuller understanding of the burden of alcohol-related injury in society. To assess such externalities, investigators for the ED studies in the WHO project also obtained data on whether the patient being treated for a violence-related injury believed the other person had been drinking. Across the 14 countries, from 14 percent to 73 percent of the victims believed that others definitely had been drinking. Based on these data, the pooled estimate for the AAF was 38.8 percent when both victim and perpetrator were considered, compared with an AAF of 23.9 percent when only the patient was considered (Cherpitel et al. 2012*a*).

## Considerations and Limitations in Estimating Risk of Injury

The data reported here on the risk of injury primarily were derived from patients’ self-reports of drinking prior to injury. Although the ED studies all estimated the patient’s BAC at the time of ED admission based on breath alcohol levels, self-reports seem to be a better measure of drinking, because in many cases a substantial period of time may have lapsed between the patient’s last drink, the injury event, and arrival at the ED. As a result, the BAC may be negative even though the patient reports drinking prior to injury. Indeed, this discrepancy has been found in an analysis of the concordance between self-reported drinking and BAC measurements in the ERCAAP and WHO studies across 16 countries (Cherpitel et al. 2007).

The studies reported here all have been conducted in EDs, rather than in trauma centers that generally treat the most serious injury cases and, consequently, are less conducive to the detailed data collection effort required in studies of alcohol and injury, unless the patient is admitted to the hospital. It is unknown how this may affect the resulting conclusions regarding the rates of the risk of injury from drinking, because the literature has been mixed regarding alcohol’s association with injury severity.

As noted earlier, some limitations also apply to the methods that have been used to estimate the risk of injury related to alcohol consumption. Case–control studies may underestimate this risk because the medical patient controls also may have drinking-related conditions. The matched-interval approach to case–crossover analyses eliminates the heaviest drinkers (i.e., those who report drinking both during the period preceding the injury and during the control period), which may lead to underestimates of the risk of injury for these drinkers. Likewise, the usual-frequency approach may underestimate the risk of injury for heavy drinkers because of the increase in expected drinking occasions for the heaviest drinkers.

In addition, when estimating risk of injury using the case–crossover approach, it is important to consider the activity in which the patient was engaged at the time of injury. For example, for a patient injured in a motor vehicle accident who had been drinking, the comparison with the control time interval only would be valid if the patient also had been in a motor vehicle during the control interval. Otherwise, the patient would not have been exposed to the risk of incurring a motor vehicle–related injury, regardless of whether he or she had been drinking. This is an important consideration in future studies that seek to examine risk of injury related to alcohol.

Lastly, the risk of injury related to drinking likely is affected by a number of individual-level characteristics such as age, gender, and risk-taking disposition, as well as by societal-level characteristics such as detrimental drinking pattern, as discussed above. Estimates of AAFs for injury, which are required for determining the global burden of disease for injury related to alcohol, generally have not taken these variables into consideration, and this is a necessary direction for future research on the burden alcohol-related injury puts on society.

## Footnotes

1 The OR is the ratio between the risk that a person with a certain characteristic (e.g., positive BAC) experiences a certain outcome (e.g., an injury) and the risk that a person without that characteristic experiences the same outcome. In other words, an OR of 2.4 indicates that people who have a positive BAC are 2.4 times as likely to be injured as people without a positive BAC.

2 As used here, the term “alcohol-related injury” refers to injuries where the patient reported using alcohol in the 6-hour period immediately preceding the injury; in contrast, the term “injury” is used here to refer to any injury, regardless of whether it was preceded by alcohol use or not.
